# Distal femoral valgus osteotomy: bone healing time in single plane and biplanar technique

**DOI:** 10.1007/s11751-016-0266-2

**Published:** 2016-10-14

**Authors:** J. A. D. van der Woude, S. Spruijt, B. T. J. van Ginneken, R. J. van Heerwaarden

**Affiliations:** 1 Limb Deformity Reconstruction Unit, Department of Orthopaedic Surgery, Maartenskliniek Woerden, Polanerbaan 2, 3447 GN Woerden, The Netherlands; 2 Centre for Deformity Correction and Joint Preserving Surgery, Kliniek ViaSana, Hoogveldseweg 1, 5451 AA Mill, The Netherlands

**Keywords:** Distal femoral osteotomy, Valgus producing, Closed-wedge, Limb alignment, Biplanar, Uniplanar

## Abstract

Varus deformity can be localized in the tibia, in the femur or in both. If varus deformity is localized within the femur, it is mandatory to correct it in the femur. This report presents the technique and results of a consecutive case series of lateral uniplanar and biplanar closed-wedge valgus osteotomy of the distal femur for the treatment of varus deformity of the knee. Retrospectively, fifteen patients (sixteen knees) were identified. Indications for surgery varied from unloading an osteoarthritic medial compartment to reduction to symmetrical varus leg alignment. Pre- and post-operative X-rays, including a full leg radiograph, were assessed as well as bone healing time at follow-up intervals. Clinical outcome was assessed using different questionnaires. There were nine male and six female patients with a median age at surgery of 45 (±14) years. The mLDFA changed from 95.9° (±2.7°) preoperatively to 89.3° (±2.9°) post-operatively. Preoperative planning and the use of angle stable implants resulted in accurate corrections according to preoperative aims in all but one patient. At follow-up (mean, 40 months), the mean VAS score was 2.5 (±2.4) and the WOMAC score averaged 80 (±20). The mean bone healing time of biplanar osteotomies (4 ± 3 months) was shorter than in the uniplanar osteotomies (6 ± 3 months). Distal lateral closed-wedge valgus osteotomy of the femur for the treatment of femoral varus deformities resulted in clinical improvement and accurate corrections in patients with different aims for correction. A biplanar osteotomy technique shortens bone healing time.

## Introduction

Varus malalignment of the knee is associated with the development and progression of knee osteoarthritis [1]. In a biomechanical study, it was demonstrated that the cartilage of the medial compartment of the knee is loaded predominantly in a varus knee; a neutral mechanical axis loads the medial slightly more than the lateral compartment; and in valgus alignment the main load is through the lateral compartment [2].

**Fig. 1:**
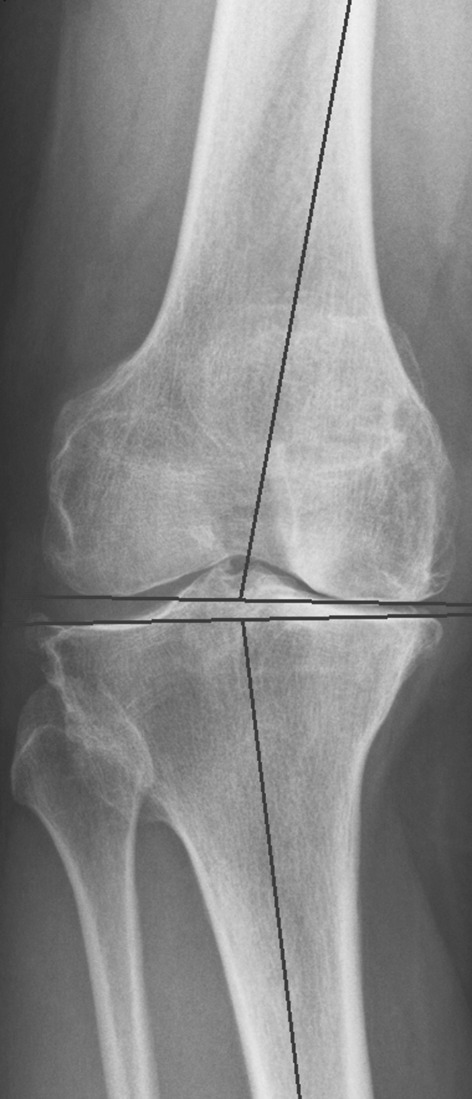
Example of varus deformity in the distal femur (mLDFA 100°, MPTA 86°)

The rationale for osteotomies around the knee in symptomatic osteoarthritic joints is to offload the affected compartment by shifting the weight-bearing axis to the more normal compartment and achieve a more even distribution of pressure and accomplish pain relief. In addition, osteotomies are indicated to correct deformity or to obtain alignment symmetrical to the contralateral side. Traditionally, a high tibial osteotomy (HTO) is used to correct varus deformity and distal femoral osteotomy (DFO) to correct a valgus deformity. However, the source of a varus deformity can be localized in the tibia, in the femur (Fig. 1) or in both. The same is true for a valgus deformity. If a varus deformity that is localized in the femur is corrected using a valgus-producing HTO, the end results will be a re-aligned limb axis at the cost of an excessively oblique joint line [3, 4]. Joint-line obliquity of the knee is not tolerated well because of increased shear stresses [3] and may lead to technical difficulties when performing a total knee arthroplasty [5].

Distal femoral osteotomy techniques for lateral OA from femoral deformities have evolved to more accurate corrections, decreased bone healing problems and improved clinical scores [6–9]. Whilst the literature on varus osteotomies on the distal femur is increasing, reports on valgus distal femoral osteotomies are scant. This retrospective review presents the technique and results of a consecutive series of lateral closed-wedge valgus osteotomies of the distal femur for the treatment of symptomatic varus deformity.

## Materials and methods

### Sample

We identified fifteen patients (sixteen knees) who underwent a closed-wedge valgus-producing osteotomy of the femur for the treatment of varus deformity in our department in the past decade. The osteotomies were performed between 2005 and 2012, in two centres in the Netherlands (Maartenskliniek Woerden and Sint Maartenskliniek Nijmegen). Two experienced surgeons (RJvH and SS) performed all osteotomies using the techniques described below. The objectives of surgery differed: indications included medial compartment offloading in medial osteoarthritis; a decrease in varus alignment to normal; and a restoration of leg alignment symmetrical to the contralateral leg.

### Radiograph measurements

All patients underwent preoperative and post-operative plain X-rays of the knee in 3 planes (AP weight-bearing view, lateral view, PA 45° weight-bearing tunnel view and patella skyline view) and a standing full leg AP radiograph. The standing full leg antero-posterior radiographs were obtained using a standardized protocol; patients stood on both feet with the knees in full extension and with the X-ray beam centred on the knee [10]. The degree of osteoarthritis was scored using Kellgren and Lawrence scale [11]. In addition, the degree of varus deformity was assessed by measuring the mechanical tibiofemoral angle, the medial proximal tibia angle (MPTA), the mechanical lateral distal femoral angle (mLDFA) and the knee joint-line convergence angle (JLCA) preoperatively and post-operatively [4]. The mechanical axis of the femur is defined as the line between the centre of the femoral head (identified using Mose circles) and the apex of the intercondylar notch of the femur. The mechanical axis of the tibia runs from the mid-point between the tibial spines to the mid-width of the distal tibia. The mechanical tibiofemoral angle is the angle between the mechanical axis of the femur and tibia [4] and was expressed as a deviation from 180° (positive values indicate varus, negative values valgus). The MPTA is the angle measured medially between the mechanical tibial axis and the tibial joint line (defined as a line tangential to the flat or concave aspect of the subchondral line of the two tibial plateaus) [4]. The mLDFA is the angle measured laterally between the femoral mechanical axis and the femoral joint line (a line tangential to the most distal points on the convexity of the two femoral condyles) [4]. MPTA and mLDFA values between 85° and 90° are considered normal. A MPTA less than 85**°** indicates that the varus deformity is located in the tibia. When there is a mLDFA higher than 90**°**, the femur contributes to the varus deformity. The JLCA was defined as the angle between the femoral and tibial knee joint lines in the frontal plane. A medially converging joint line greater than 3° is abnormal and indicates either ligamentous laxity or loss of cartilage thickness as source of varus malalignment [4]. All measurements were performed by two of the authors (SS and JTW). There were no cases of joint contractures to influence radiographic measurements.

### Clinical outcome

The range of motion of the knee was measured preoperatively and during post-operative visits. Knee joint function and quality of life (Qol) were evaluated post-operatively using the validated Dutch knee injury and osteoarthritis outcome score (KOOS) [12] and the Western Ontario and McMaster Universities Osteoarthritis Index (WOMAC) [13], both normalized to a 100 % scale, 100 being the maximum score. The VAS pain score (0–100 mm; “0” meaning no pain) was used to evaluate pain. The Lysholm knee score provided information on instability and functional limitations [14], and the Tegner knee function score (range 0–10) was used to determine the level of activity in work and sports [15]. Questionnaires were sent by postal mail to all patients.

### Operative technique

Surgery is performed in supine position with the knee in full extension, and a tourniquet is placed at the root of the thigh to create a bloodless field. A single dose of antibiotic is used preoperatively. Fluoroscopic visualization of the hip, knee and ankle joint is used during surgery.

A 10–15 cm straight lateral incision is made, starting 3 cm proximal to the knee joint line and extending proximally. With the fascia lata split longitudinally, a lateral subvastus approach is started by palpation of the natural opening under the distal part of the vastus lateralis muscle belly at the level of the supratrochlear area. A retractor is used to lift the muscles anteriorly. The dorsal part of the lateral vastus muscle is freed from the intermuscular septum by blunt and sharp dissection. Special care is taken to visualize and ligate the perforating vessels present whilst creating enough room proximally for plate fixation. A blunt Hohmann retractor is placed posteriorly in contact with the bone to protect the popliteal neurovascular bundle.

**Fig. 2:**
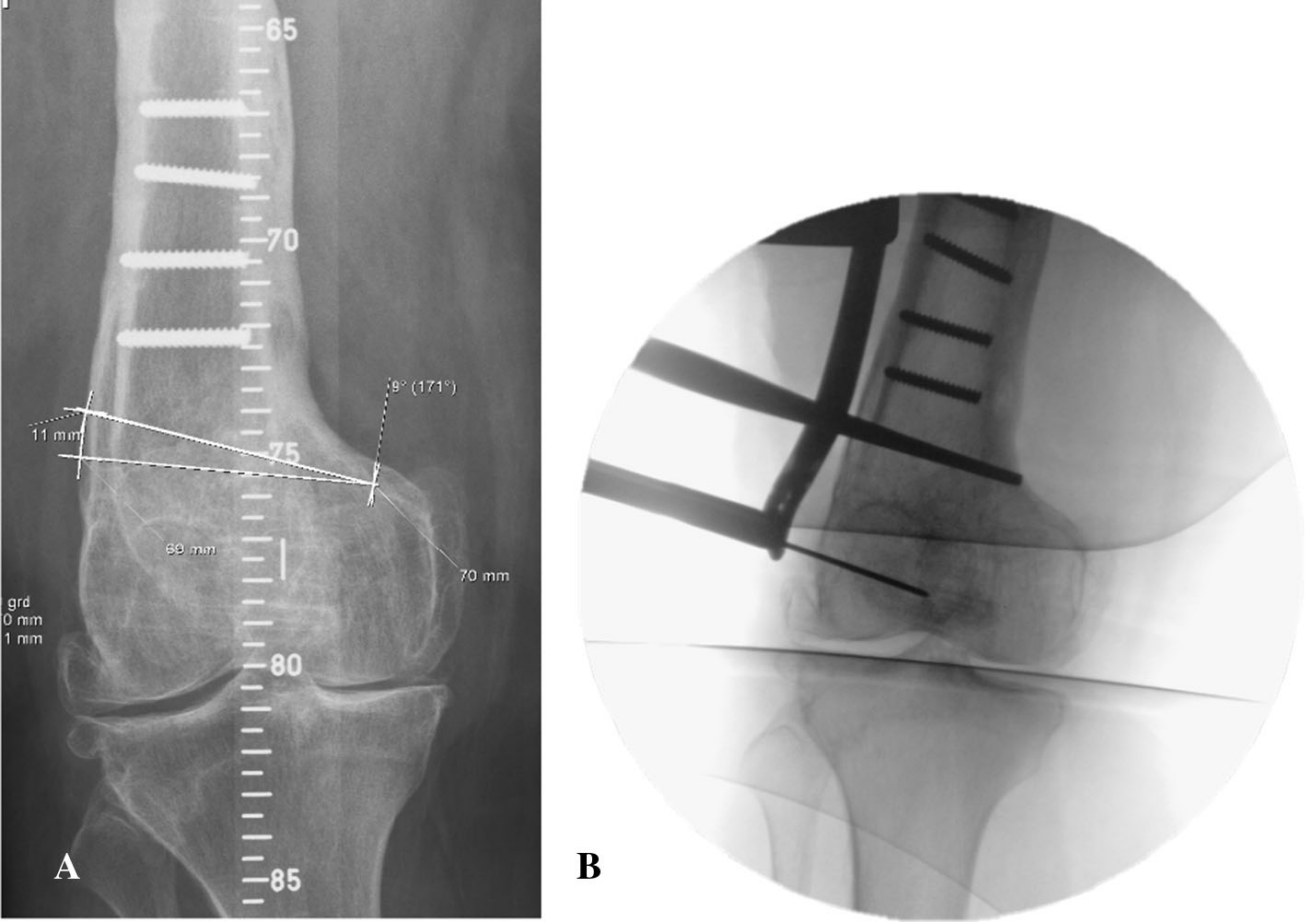
The starting point for the distal osteotomy at the lateral femur is defined by preoperative digital planning (**a**) and intraoperative fluoroscopy check using temporary plate application (**b**) to relate osteotomy height to optimal plate position

The starting point for the distal osteotomy on the lateral femur is determined through preoperative digital planning and an intraoperative fluoroscopy check using temporary plate application to locate osteotomy level to optimal plate position (Fig. 2) The desired level of the osteotomy is marked. Under fluoroscopic control, two K-wires are inserted for an oblique down-sloping wedge with the wedge base length at the lateral cortex corresponding to the preoperative planning. The K-wires converge just proximal to the medial femoral condyle, ending 0.5–1 cm short of the medial cortex, and may be inserted freehand or with an osteotomy guide.

**Fig. 3:**
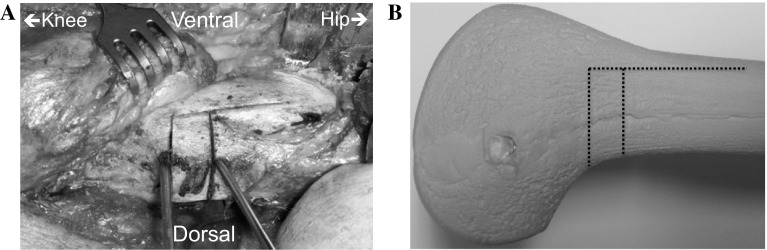
Example of the biplanar technique in a left distal femur intraoperatively (**a**) and in a sawbone (**b**). The two transverse cuts are made in the dorsal three-fourth, whereas the proximal directed frontal plane saw cut is made in the ventral one-fourth of the distal femur

A uniplanar closing-wedge osteotomy was performed between 2005 and 2008 by making two transverse cuts with an oscillating saw within the two K-wires. After 2009, we used a biplanar osteotomy technique [16]. In the biplanar technique, the dorsal three-fourth is used for the two transverse osteotomy cuts, whereas a proximally directed frontal plane saw cut is made in the ventral one-fourth of the distal femur. The dorsal cortex is used as a reference for directing the frontal plane cut across the ventral surface; this is performed with a thinner saw blade (Fig. 3).

After wedge removal, the resected wedge is inspected for completeness as remaining bone fragments may cause incomplete closure and fracture of the medial cortical hinge during closure. If this is found, additional bone removal and weakening of the hinge (with help of a special bone impaction instrument—a blunt chisel) are then indicated. Closure of the wedge must be performed gradually and with a gentle valgus force. It may take several minutes to enable plastic deformation of the medial cortex to close the osteotomy gap. It should be noted that the medial cortex of the distal femur in general is weaker and the hinge point of the osteotomy will fracture more often as compared to the lateral cortex hinge point in a medial closing-wedge osteotomy. An intact medial cortex after osteotomy closure provides for higher axial and rotational stability.

Limb alignment is evaluated fluoroscopically using a long rigid alignment rod between the centre of the femoral head and the centre of the ankle. The rod, representing the weight-bearing line, should pass through the knee joint at the preoperatively defined position for the mechanical axis. If adequate correction has been achieved, the osteotomy is stabilized with either a TomoFix (Depuy/Synthes) Lateral Distal Femur plate (LDF) (ipsilateral version) or with a TomoFix Medial Distal Femur Plate (MDF) (contralateral version). The decision is based on personal choice of the surgeon. However, the MDF plate is less pronounced after insertion and therefore more suitable in shorter and smaller femurs.

The plate is mounted with drill guides and is, with a spacer to protect the periosteum, distally placed on the lateral femur condyle and proximally in line with the femur shaft in the frontal and sagittal plane. Temporary fixation distal to the osteotomy is performed with a K-wire drilled through a guiding sleeve. Plate position is checked fluoroscopically. As the TomoFix is an internal fixator, precise fit to the femur is not necessary. After drilling, at least four self-tapping locking screws are inserted distally. Next, a bicortical self-tapping lag screw is inserted eccentrically in the dynamic part of the combi-hole directly superior to the osteotomy putting the osteotomy under axial compression. Three self-tapping monocortical or bicortical (depending on bone quality and patient’s stature) screws are inserted in the remaining holes proximal of the lag screw. Finally, the lag screw is changed for a self-tapping bicortical locking screw inserted in the locking part of the combi-hole. After a final check with the image intensifier, the wound is closed over a non-suction drain. Care is taken to meticulously close the fascia lata before subcutaneous closure. The skin is closed subcuticularly.

### Post-operative care

A sterile compressive bandage is applied after surgery. In the first 24 h during rest, the knee is positioned in a 60–90° flexion position to prevent adhesions of the vastus lateralis muscle to the femur [17, 18]. Full range of active and passive movement of the knee is started as soon as tolerated by the patient with the help of a physiotherapist. During the first 6 weeks partial (no more than 15–20 kg) weight-bearing is allowed between crutches. Clinical and radiographic proof of bone healing at 6 weeks enables progressive weight-to-full-weight bearing.

### Comparison of bone healing time

Bone healing at 6 weeks, 3 months, 6 months, 9 months and 12 months post-surgery was evaluated on standard coronal and sagittal radiographs. Full bone healing was defined as full reformation, though osteotomy recognizable, as described by van Hemert et al. [19] Bone healing time at different follow-up times for biplanar and uniplanar osteotomies was scored and compared using standard *T* test for comparison.

## Results

Of the fifteen patients (sixteen knees) who underwent an isolated valgus-producing closing-wedge distal femoral osteotomy (DFO), one patient had a total knee arthroplasty within 2 years. There were nine male and six female patients with a median age at surgery of 45 (±14) years. Preoperatively, 63 % of the cases had a Kellgren and Lawrence grade of III. Table 1 shows the sample characteristics. One patient had a bilateral closed-wedge valgus DFO. The causes of varus deformity were: femoral malunion in five knees; overcorrection of a valgus deformity (previous osteotomy) in four knees; secondary to an (hemi)-epiphysiodesis in two knees; and idiopathic in five knees with osteochondritis dissecans of the medial femoral condyle in two knees. Five osteotomies were preceded by an arthroscopy; one had a partial lateral meniscectomy and four a partial medial meniscectomy.

**Table 1:** Characteristics of the study population

Number of patients (*n*)	15
Number of osteotomies (*n*)	16
Mean age at surgery [years (±SD)]	45 ± 14
Gender ratio (M:F)	9:6
Mean body length at surgery [cm (±SD)]	180 ± 11
Mean weight at surgery [kg (±SD)]	86 ± 20
Mean body mass index at surgery [kg/m^2^ (±SD)]	26 ± 4
Side (left:right)	6:10
Kellgren and Lawrence grade	
Grade 1 [*n* (%)]	2 (12.5 %)
Grade 2 [*n* (%)]	3 (18.8 %)
Grade 3 [*n* (%)]	10 (62.5 %)
Grade 4 [*n* (%)]	1 (6.3 %)
Mean follow-up [months (±SD)]	40 ± 30

### Operative data

There were no intraoperative complications. The mean duration of the surgery was 89 min (range 50–135 min). In six knees, the DFO was uniplanar and in ten biplanar. An angular stable LDF plate was used in twelve knees, an angular stable MDF plate (contralateral) in three and in one knee, because of non-availability of other plates at time of surgery, a LISS plate. In two knees, additional fixation was used: in one knee a staple at the fractured medial hinge and in one other knee an antero-posterior lag screw through the anterior flange of the biplane osteotomy. A fracture of the hinge without dislocation was observed in eight knees.

No systemic complications, wound infections, or nerve palsies occurred. Due to tenderness, seven patients required plate removal. In one patient, an ACL-reconstruction as well as an open-wedge valgus high tibial osteotomy was performed several years after the index surgery for progressive symptomatic medial osteoarthritis causing tibial varus deformity and instability. In two patients, an arthroscopy was necessary (amongst them the patient who underwent the total knee arthroplasty).

### Radiographic measurement results

**Fig. 4:**
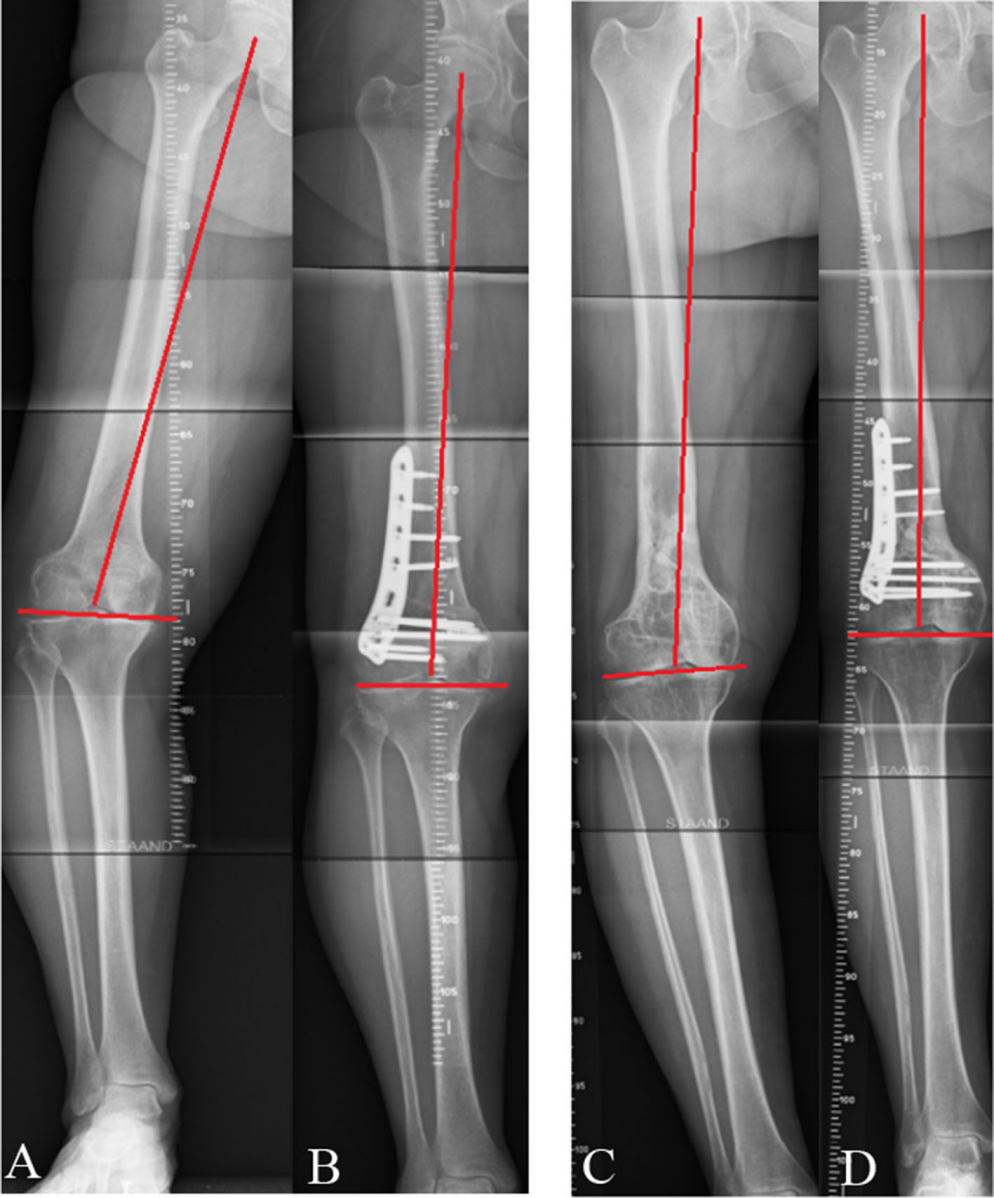
Leg alignment preoperative (**a**–**c**) and 3 months post-operative in two cases (**b**–**d**)

**Fig. 5:**
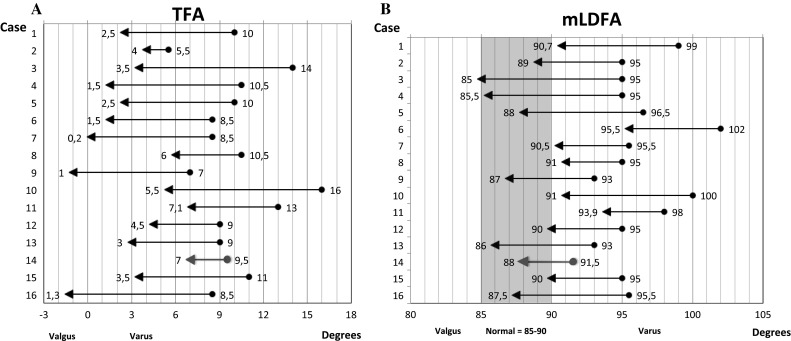
Change of mechanical tibiofemoral angle (TFA) per patient (**a**) and the change of mechanical lateral distal femoral angle (mLDFA) per patient (**b**). The preoperative deformities are represented by the *circles* and the post-operative values are represented by the *arrowheads*. The *red line* represents the failure (i.e. total knee arthroplasty) (color figure online)

The mean preoperative mechanical tibiofemoral axis was 10.0° (±2.6°) of varus which reduced to 3.1° (±2.6°) varus after surgery. The mLDFA changed from 95.9° (±2.7°) preoperatively to 89.3° (±2.9°) post-operatively. The mean MPTA did not substantially contribute to varus in this group of patients, being 87.8° (±2.3°) preoperatively. Figure 4 shows pre- and post-operative leg alignment in two cases. All pre- and post-operative radiographic measurements are in Table 2 and Fig. 5. The preoperative indication and aim of correction of each case are displayed in Table 3.

**Table 2:** Preoperative and post-operative radiographic measurements

Preoperative						Post-operative					
Case	K&L	TFA	MPTA	mLDFA	JLCA	K&L	TFA	mLDFA	JLCA	HF	BHT
1	3	10	90	99	2	4	2.5	90.7	1.4	Yes	9
2	4	5.5	*92*	95	3.5	4	4.0	89	2.5	No	3.8
3	3	14	86	95	5	3	3.5	85	5.5	Yes	4.8
4	1	10.5	86.5	95	3	1	1.5	85.5	3.5	No	3.5
5	2	10	87.5	96.5	2	2	2.5	88	2	Yes	3
6	3	8.5	93	102	0.5	3	1.5	95.5	2 Lat	Yes	3
7	2	8.5	89	95.5	3	2	0.2	90.5	0.5 Lat	No	5.5
8	3	10.5	88	95	4	3	6	91	5	Yes	10
9	3	7	88	93	2	3	−1	87	1.5	No	2.3
10	3	16	86	100	2.5	3	5.5	91	l Lat	Yes	2.3
11	2	13	86	98	0.5	2	7.1	93.9	1.4	Yes	7
12	3	9	88.5	95	3	3	4.5	90	3	No	1.5
13	3	9	86	93	2	3	3	86	3	Yes	8
14	3	9.5	87	91	5	3	7	88	5	No	7
15	3	11	85	95	1.5	3	3.5	90	0	No	4
16	1	8.5	86	95.5	0.5 Lat	1	−1.3	87.5	1 Lat	No	1.5

K&L scale of Kellgren and Lawrence, grade 0 normal, grade 1 min osteophytes, grade 2 definite osteophyte, grade 3 moderate joint-space reduction, grade 4 severe joint-space narrowing with sclerosis and osteophytes, TFA mechanical tibiofemoral angle (degree, positive values indicate varus alignment, negative values indicate valgus alignment), MPTA medial proximal tibial angle (degree)

*mLDFA* mechanical lateral distal femoral angle (degree), *JLCA* joint-line convergence angle (degree), *Lat* lateral convergence, *HF* hinge fracture, *BHT* bone healing time (months)

**Table 3:** Indication, aim of correction, clinical scores and plate complaints

Preoperative			Post-operative				
Case	Ind.	Aim	VAS	WOMAC	Lys	Teg	PC
1	PO	B	1	81	73	2	No
2	ID	A	5	74	63	5	No
3	PE	C	1	93	82	2	No
4	PT	B	2	99	92	7	Yes
5	PT	B	0	100	85	3	Yes
6	PO	B	0	92	80	2	No
7	ID	A	7	21	32	0	Yes
8	OCD/ID	A	2	75	58	3	Yes
*9*	OCD/ID	A	2	75	58	3	Yes
10	PE	C	3	57	78	2	No
11	PT	B	1	98	97	5	No
12	PO	B	7	76	67	2	No
13	ID	A	–	84	60	2	No
14	PT	A	–	–	–	–	Yes
15	PT	B	4	81	75	3	Yes
16	PO	A	2	94	90	3	No

*Ind.* indication, *PT* post-traumatic (femoral malunion), *PE* previous epiphysiodesis, *ID* idiopathic, *PO* previous osteotomy, *OCD* osteochondritis dissecans, Aim: A unloading, B correction to normal varus, C correction to symmetrical leg alignment, Lys Lysholm, Teg Tegner, PC plate complaints resulting in plate removal. Case 14 represents the failure (i.e. total knee arthroplasty)

### Clinical results

As one patient had a total knee arthroplasty, fourteen (fifteen knees) of the included fifteen patients (sixteen knees) could be evaluated clinically (Table 3). The clinical results were assessed at a mean of 40 months (±30) post-operatively. At follow-up, the mean VAS score was 2.5 (±2.4). The subjective result according to the Lysholm score was excellent in one patient, good in three patients, fair in six patients and poor in four patients. All the patients who scored good or excellent on the Lysholm scale had grade I or II of osteoarthritis according to the scale of Kellgren and Lawrence. On the Tegner activity scale, the mean level was 3 (±1.7). At follow-up, the WOMAC score averaged 80 (±20). The mean score at follow-up of the individual components of the WOMAC index (pain, stiffness and function) were 80 (±18), 75 (±26) and 81 (±21), respectively. The range of flexion and extension did not change between preoperative and post-operative measurements (118° ± 14° preoperative versus 117° ± 15° post-operative). The mean length of hospital stay was 3 (±1) days.

### Bone healing time results

All but 3 patients in the biplane DFO group showed union at the 3 months follow-up radiographs. The remaining patients showed union at, respectively, 6, 7 and 9 months of follow-up. In the single plane DFO group, two patients showed union at the 3 months follow-up radiographs, one at 5 months, one at 7 months, one at 8 months and one at 10 months. Comparison of the mean time to union between the biplane osteotomy group (3.9 ± 2.5 months) and the single plane group (6.1 ± 2.7 months) did not show a significant difference (*p* = 0.118).

## Discussion

This retrospective cohort study is a report on the short- to mid-term results of the distal lateral closed-wedge valgus osteotomy of the femur. Carefully planned single plane and biplane osteotomies have produced significant symptom relief in most patients although clinical scores in two patients indicated persistent functional impairments.

Deformities around the knee should be subject to a systematic deformity analysis using standardized full leg standing radiographs [20]. In this sample, a femoral deformity was identified as the origin of the varus of the leg. Each deformity should be corrected at its source otherwise joint-line obliquity will be the result [5, 21]. Accordingly, valgus osteotomies are performed at the tibial level, femoral level or both levels simultaneously depending on the source of the deformity (i.e. a tailored approach) [3]. Joint-line obliquity is to be avoided as it results in increased shear stresses at the cartilage joint surface (even tibiofemoral subluxation) and may hamper subsequent joint replacement surgery.

The influence of joint-line obliquity and varus orientation of the distal femur on the results of osteotomies around the knee has been reported. Terauchi et al. [22] found that the presence of a preoperative varus deformity of the distal femur was associated with recurrence of varus deformity and poor results after HTO. Van Raaij et al. [23] did not find a significant correlation between distal femoral joint-line orientation and failure of HTO. This can be explained by the fact that the mean preoperative distal femur alignment in their patients was mild valgus (mean mLDFA 89.1 ± 2.1°), whereas our patients had a clear varus malalignment of the distal femur with a mean mLDFA of 95.9° (±2.7°). Babis et al. [5] looked at obliquity of the joint line as a prognostic factor. In a series of patients with large varus deformities and medial compartment osteoarthritis, treated with a double level osteotomy, normal knee joint-line orientation was preserved and they showed in a computer model that the tension of stabilizing ligaments (i.e. collateral ligaments) remained normal after correction.

The leg alignment after deformity correction ranged from 1.3° valgus to 7.1° varus; the aims for correction differed from unloading in case of medial compartment osteoarthritis, decrease in varus to normal varus or restoration of limb symmetry (see also Table 3). In four patients, the valgus osteotomies were performed for a varus that had arisen from a previous overcorrection of a valgus deformity. In most of these cases, a neutral mechanical axis was intended. One osteotomy had resulted in an under correction. Performing a closed-wedge osteotomy is known to be difficult technically because the surgeon has to rely on the accuracy of the bone resection. Careful preoperative planning and the use of oblique osteotomy cuts of equal length in an isosceles triangle prevent cortical overlap after gap closure [6, 16]. Our final range of tibiofemoral angles were within a similar range to that published for distal femoral varus osteotomies (6° varus to 10° valgus) [24].

Our rate of hinge fractures (50 %) (Table 2) is high compared with the 10–20 % reported after closing-wedge HTO [25]. One of the main reasons for this difference may lie in the correction. For example, in six of the sixteen osteotomies the correction angle was greater than 8°; the risk of a hinge fracture gets higher when the correction angle increases due to the limited plasticity of the cortical (supracondylar) bone [26]. None of the fractured hinges displaced and, by using a temporary bicortical lag screw compression over the osteotomy, including the hinge, stability was restored. In those patients who had more developed leg muscles (and were thought to expose the osteotomy to more axial and torsional loading), a medially placed staple was used or an antero-posterior lag screw through the anterior flange of the biplane osteotomy.

The highest clinical scores were found in patients with post-traumatic deformities that according to aim had been corrected to normal varus alignment (Tables 2, 3). Patients with a failed previous femoral osteotomy had high clinical scores also, whereas lower scores were found in patients presenting with grade III osteoarthritis following osteochondritis dissecans (cases 8 and 9). In our sample, eleven osteotomies were performed in patients with moderate and severe (stage III and IV) osteoarthritis according to the scale of Kellgren and Lawrence. As observed by other authors a significant association exists between preoperative Kellgren and Lawrence grade and HTO failure [27]. There were moderate results in these patients with an average WOMAC score of 80 and only one patient requiring a total knee arthroplasty. It should be noted that in a femoral realignment osteotomy, axis restoration is planned and accomplished for the extended knee (i.e. walking). In 90° of flexion, the contact point of the loaded posterior condyles on the tibia remains unchanged [6].

One case (6.3 %) required a total knee arthroplasty and was classified as a failure. This is in line with failure rates of HTO (3.4 % before 24 months to 7.8 % between 24 and 47 months [25]) and double level osteotomy (3.7 %) [5]. In hindsight, this patient might not have been the ideal candidate for a closed-wedge valgus DFO. In this case, the aim was to correct the femoral deformity with unloading the OA. The preoperative Kellgren and Lawrence grade was III, the mLDFA was not that abnormal (91.5°) preoperatively and the post-operative mechanical tibiofemoral axis was 7° of varus. In seven cases (44 %), the fixation implant was removed. Jacobi et al. [28] reported that fixation of an osteotomy on the lateral side of the distal femur leads to irritation of the iliotibial band. Nevertheless, our rate of 44 % is lower than the 86 % of Jacobi et al. [28]. The lower rate of plate irritation in our sample may be due to the use of the less prominent MDF plate for fixation. None of the three patients with a MDF plate needed removal.

After the introduction of a biplanar technique in medial closing-wedge distal femur osteotomies [8] in our group, a biplanar osteotomy technique was used for lateral closing-wedge osteotomies. Clinical observations and demonstrations in sawbone models would suggest that a biplane medial closing-wedge osteotomy has better bone healing potential over a uniplanar technique [29]. In clinical studies, rapid and uncomplicated bone healing has been found using biplanar osteotomies in medial closing-wedge osteotomies [9] as well as for lateral opening-wedge [30] osteotomies. Bone healing time of the uniplanar osteotomies in the present study was 6.1 ± 2.7 months, whereas the bone healing time of the patients operated with a biplanar technique averaged 3.9 ± 2.5 months. Bone healing was complete in 7 of 10 patients operated on with the biplanar technique at the 3-month follow-up; this is comparable to the bone healing times reported for uniplanar [7, 8] and biplanar medial closing-wedge distal femoral techniques [9] and to those for the lateral open-wedge biplanar osteotomy results of Bagherifard et al. [30]. Of the remaining 3 patients with longer bone healing times in the biplanar osteotomy group, 2 had medial hinge fractures. Increased bone healing time from hinge fractures causing instability in closing-wedge osteotomies has been reported for DFO and HTO [31, 32]. In our population, the mean bone healing time in patients with hinge fractures was 5.8 ± 2.8 months.

Our study has limitations. It was a retrospective study with a small sample. Due to this limited number of patients, the correlation of different variables was not possible. The next step would be a prospective study comparing patients preoperatively and post-operatively after a distal lateral closed-wedge valgus osteotomy of the femur. Nevertheless, the results in our series are encouraging for selected knees. Regarding bone healing time evaluation, the intervals of follow-up hampers an accurate registration of bone healing time. A monthly follow-up would have given us more accurate information on bone healing time.

Based on the results of this study, a biplane distal lateral closed-wedge valgus osteotomy of the femur for the treatment of varus deformity of the knee is a valuable procedure when the deformity is localized in the femur with clinical benefit in most of the patients.
